# Reply to ‘Forward models of repetition suppression depend critically on assumptions of noise and granularity’

**DOI:** 10.1038/s41467-020-18316-9

**Published:** 2020-09-18

**Authors:** Arjen Alink, Hunar Abdulrahman, Richard N. Henson

**Affiliations:** 1 University Medical Center Hamburg-Eppendorf, Hamburg, Germany; 2 Medical Research Council, Cognition and Brain Sciences Unit, University of Cambridge, Cambridge, Cambridgeshire UK

**Keywords:** Perception, Neural encoding

**Replying to** F. M. Ramírez & E. P. Merriam *Nature Communication*s 10.1038/s41467-020-18315-w (2020)

In their commentary on our paper, Ramírez and Merriam^1^ make an important point that our conclusion that a local scaling model best explains the features of functional magnetic resonance imaging (fMRI) repetition suppression^2^ depends on the signal-to-noise ratio (SNR). We accept this point, and were remiss not to explore a wider range of SNRs. However, we have now run further simulations and find that, while the main alternative model—the local sharpening model—can fit the data from our Experiment 2 if the SNR is high enough, it cannot fit the data from our Experiment 1 (which Ramírez and Merriam ignored) across any SNR that we explored.

Our discussion with Ramírez started when he discovered an error in the simulation code that we made available with our paper. In our paper, we stated that the simulated fMRI noise (which is added to the voxel patterns for each simulated trial) was drawn independently and randomly for each voxel from a zero-mean Gaussian distribution, scaled to have a standard deviation of 0.1 (a parameter that Ramírez and Merriam call *σ*_Noise_). This corresponds to the Matlab (release 2012b, https://www.mathworks.com/) command “randn”, yet we inadvertently typed the Matlab command “rand” instead, which draws values randomly from a uniform distribution from 0 to 1. When the code is corrected to “randn”, *σ*_Noise_ needs to be smaller to reproduce our results (because the spread of values obtained from “randn” is ~3.5 times greater than that produced by “rand”). Indeed, when we re-run the models using Gaussian *σ*_Noise_ = 0.1/3.5–0.029, we re-confirmed that only the local scaling model meets the six empirical criteria described in our paper for both of our two experiments. This is to be expected because the analytical form of the noise distribution should not affect the qualitative pattern of results.

Ramírez and Merriam note that, if the signal in the model (for a single voxel) were 1 and *σ*_Noise_ = 0.029, and SNRs were measured as the ratio of signal power to noise power (variance), then the simulated SNR would be nearly 1200, which would be unlikely for fMRI. However, this figure for SNR is wrong because, as Ramírez and Merriam also note, the signal in the model depends on the number of neuronal populations per voxel (what they call “granularity”, and what we defined as *N*), the tuning width of each population (which we called *σ*), as well as the stimulus preference for each population (which we called *µ*). Our original paper assumed eight neural populations per voxel (i.e., *N* = 8), with stimulus preferences (*µ* values) that were randomly, uniformly distributed across the stimulus dimension *0–X*. The resulting mean simulated signal across voxels for initial presentations of stimuli in our Experiment 2 (on which Ramírez and Merriam focused) ranged from 0.1 to 0.9, with a typical value usually much less than 1.

Ramírez and Merriam also note that we did not explore the effects of different numbers of neural populations per voxel, that is, explore a range of granularity values, viz our parameter *N*. We acknowledge that changing *N* not only changes the magnitude of the signal in the model (as above), but could also affect the nature of that signal, that is, the type of multivariate patterns across voxels^3,4^. Therefore, we have re-ran simulations in which we varied both the SNR, via the noise parameter *σ*_Noise_, and the granularity, via our *N* parameter. In other words, we ran an exhaustive grid search over plausible values for these two parameters, for each model and experiment, in addition to the original ranges of values for the two to three parameters specific to each model (as in our main paper). For our Experiment 2 (using visual gratings), we confirmed Ramírez and Merriam’s observation that the local sharpening model can fit the data in addition to the local scaling model (but none of the other models could), at an SNR higher than what we originally simulated. However, the SNR needed for the local sharpening model to fit the data features was higher than for the local scaling model (~89 versus ~32), and entailed fewer sets of parameter values that allowed such a fit (see Fig. 1). More importantly, for Experiment 1 (using faces), only the local scaling model could fit the data features (at SNRs of ~5). In other words, while the local sharpening model could fit the data from one experiment, it could not fit the data from both experiments—an observation that Ramírez and Merriam choose not to highlight in their commentary. Thus, we stand by our original claim that only local scaling is consistent with our data, as now confirmed across a range of SNRs and a range of granularities.

**Fig. 1:**
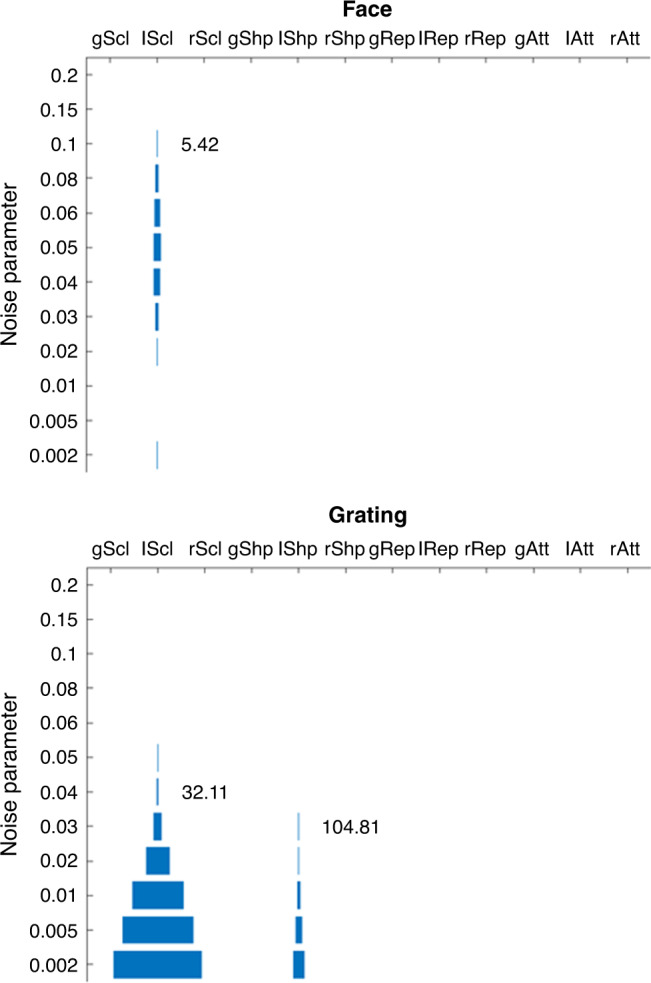
The effect of noise on model fits. This figure shows which models (*x*-axis) could fit the data from each experiment as a function of the noise parameter (*y*-axis) after an exhaustive grid search of values for five parameters (σ_Noise_, *N*, *a*, *b* and *σ*; see text). The width of the horizontal bars reflects the number of unique sets of parameter values that could fit the data patterns for each model (for a given noise level). The number to the right of bars shows the lowest signal-to-noise ratio (SNR) achievable (at the location of the corresponding noise parameter value). Note that the SNR is related to the noise parameter, but also depends on the other model parameters, which affect the signal, so does not necessarily occur at the lowest noise parameter value. The range of parameter values explored were *N* = [4 8 16 32], *a* = [0.1 0.2 0.3 0.4 0.5 0.6 0.7 0.8 0.9], *b* = [0.1 0.3 0.5 0.7 0.9 1.1 1.3] and *σ* = [0.1 0.3 0.5 0.7 0.9 2 4 6 8 10] (unique noise parameter values shown on *y*-axis). The important point is that only the local scaling model could fit the face data (up to SNRs of 5.42), and while local sharpening could also fit the grating data, as Ramirez and Merriam noted, it required a higher SNR (104.81) and did so with fewer sets of parameter values. Acronyms on the *x*-axis stand for (from left to right) the following models: global scaling, local scaling, remote scaling, global sharpening, local sharpening, remote sharpening, global repulsion, local repulsion, remote repulsion, global attraction, local attraction and remote attraction.

Ideally, we would select an SNR for the models that matches that in the data. However, estimating the SNR of fMRI data is nontrivial. SNR is normally expressed in terms of the original fMRI time-series, for example, from the variance of the fitted response (using a General Linear Model (GLM)) relative to the variance of the residuals of the fit. However, our model operates at the level of patterns of responses across voxels for each trial, rather than the original fMRI time-series, so SNR must be defined across single-trial GLM estimates. These estimates have a complex noise structure, which includes sequential correlations across trials owing to the type of GLM estimation^5^ and spatial correlations across voxels. Furthermore, different SNR estimates are relevant for different data features. For example, spatially correlated noise has little effect on the mean signal across voxels (e.g. our “MAM” criterion), but has dramatic effects on classification performance based on patterns across voxels^5^, for example, our “CP” criterion. Modelling this complex noise structure requires many more parameters than our simplistic assumption of independent, Gaussian noise (with a single scaling parameter) and would be an important topic for future work. Indeed, future work could attempt quantitative rather than qualitative fitting of the fMRI data (by augmenting the models with additional scaling and noise parameters), and then use measures of model evidence to favour certain models, taking into account potential differences in the complexity of those models. For example, it is possible that the local scaling model is more complex than the local sharpening model, although we think this is unlikely, given that it has the same number of parameters, and an as simple, if not simpler, functional form.

In summary, we agree with Ramírez and Merriam that SNR and granularity are important considerations for forward modelling of fMRI data, and we have now explored a range of values for them. We find that the range of SNRs at which the local scaling model fits all our data features is larger than its main competitor—the local sharpening model. Indeed, the local sharpening model could not fit the data from our face experiment (Experiment 1) at any noise levels (or granularities) that we tried. We therefore maintain that the local scaling model is currently the most parsimonious explanation of fMRI repetition suppression. Note, however, that we were careful to point out that our modelling does not refute the single-cell evidence that adaptation can have other effects on neural tuning curves (like the attraction/repulsion models we considered), and it remains possible that a combination of neural mechanisms occurs within or between different brain regions and paradigms. Most importantly, we stand by the main methodological point of our original paper: that explicit forward models are needed in order to relate fMRI data to underlying neural models, otherwise researchers fall into the trap of thinking that an fMRI finding like improved multi-voxel classification following repetition implies that the underlying neural representations have been sharpened.

## Reporting summary

Further information on research design is available in the Nature Research Reporting Summary linked to this article.

## Supplementary information

Reporting Summary

## Data Availability

The code for our new simulations has been updated in the OSF repository cited in our original paper (https://osf.io/ph26y/). The code for the grid search of SNRs is in “ExploringSNR” folder.
