# Supplementary material for: Reply to ‘Forward models of repetition suppression depend critically on assumptions of noise and granularity’
Source: Nat Commun. 2020 Sep 18;11:4735. doi: 10.1038/s41467-020-18316-9 (PMC7501275; doi:10.1038/s41467-020-18316-9)
Supplement: Supplementary file 1 — Reporting Summary [file 41467_2020_18316_MOESM1_ESM.pdf]

nature research

Corresponding author(s): Arjen Alink

Last updated by author(s): Jun 30, 2020

Reporting Summary

Nature Research wishes to improve the reproducibility of the work that we publish. This form provides structure for consistency and transparency in reporting. For further information on Nature Research policies, seeAuthors & Referees and theEditorial Policy Checklist .

Statistics

For all statistical analyses, confirm that the following items are present in the figure legend, table legend, main text, or Methods section.

n/a	Confirmed	
☐	☒	The exact sample size (*n*) for each experimental group/condition, given as a discrete number and unit of measurement
☐	☒	A statement on whether measurements were taken from distinct samples or whether the same sample was measured repeatedly
☐	☒	The statistical test(s) used AND whether they are one- or two-sided<br>*Only common tests should be described solely by name; describe more complex techniques in the Methods section.*
☒	☐	A description of all covariates tested
☐	☒	A description of any assumptions or corrections, such as tests of normality and adjustment for multiple comparisons
☐	☒	A full description of the statistical parameters including central tendency (e.g. means) or other basic estimates (e.g. regression coefficient) AND variation (e.g. standard deviation) or associated estimates of uncertainty (e.g. confidence intervals)
☐	☒	For null hypothesis testing, the test statistic (e.g. *F*, *t*, *r*) with confidence intervals, effect sizes, degrees of freedom and *P* value noted<br>*Give P values as exact values whenever suitable.*
☒	☐	For Bayesian analysis, information on the choice of priors and Markov chain Monte Carlo settings
☒	☐	For hierarchical and complex designs, identification of the appropriate level for tests and full reporting of outcomes
☒	☐	Estimates of effect sizes (e.g. Cohen's *d*, Pearson's *r*), indicating how they were calculated

Our web collection on statistics for biologists contains articles on many of the points above.

Software and code

Policy information about availability of computer code

Data collection	N/A, see original publication
Data analysis	Matlab (Release 2012b, https://www.mathworks.com/)

For manuscripts utilizing custom algorithms or software that are central to the research but not yet described in published literature, software must be made available to editors/reviewers. We strongly encourage code deposition in a community repository (e.g. GitHub). See the Nature Research guidelines for submitting code & software for further information.

Data

Policy information about availability of data

All manuscripts must include a data availability statement. This statement should provide the following information, where applicable:

- Accession codes, unique identifiers, or web links for publicly available datasets
- A list of figures that have associated raw data
- A description of any restrictions on data availability

see original publication, and in the our response: "The code for our new simulations has be updated in the OSF repository cited in our original paper "

Field-specific reporting

Please select the one below that is the best fit for your research. If you are not sure, read the appropriate sections before making your selection.

☒ Life sciences ☐ Behavioural & social sciences ☐ Ecological, evolutionary & environmental sciences

For a reference copy of the document with all sections, see nature.com/documents/nr-reporting-summary-flat.pdf

Life sciences study design

All studies must disclose on these points even when the disclosure is negative.

Sample size	N/A, see original publication
Data exclusions	N/A, see original publication
Replication	N/A, see original publication
Randomization	N/A, see original publication
Blinding	N/A, see original publication

Reporting for specific materials, systems and methods

We require information from authors about some types of materials, experimental systems and methods used in many studies. Here, indicate whether each material, system or method listed is relevant to your study. If you are not sure if a list item applies to your research, read the appropriate section before selecting a response.

Materials & experimental systems

n/a	Involved in the study	
x		Antibodies
x		Eukaryotic cell lines
x		Palaeontology
x		Animals and other organisms
	x	Human research participants
x		Clinical data

Methods

n/a	Involved in the study	
x		ChIP-seq
x		Flow cytometry
	x	MRI-based neuroimaging

Human research participants

Policy information about studies involving human research participants

Population characteristics	N/A, see original publication
Recruitment	N/A, see original publication
Ethics oversight	N/A, see original publication

Note that full information on the approval of the study protocol must also be provided in the manuscript.

Magnetic resonance imaging

Experimental design

Design type	N/A, see original publication
Design specifications	N/A, see original publication
Behavioral performance measures	N/A, see original publication

Acquisition

Imaging type(s)	N/A, see original publication
Field strength	N/A, see original publication
Sequence & imaging parameters	N/A, see original publication
Area of acquisition	N/A, see original publication

Diffusion MRI ☐ Used ☒ Not used

Preprocessing

Preprocessing software	N/A, see original publication
Normalization	N/A, see original publication
Normalization template	N/A, see original publication
Noise and artifact removal	N/A, see original publication
Volume censoring	N/A, see original publication

Statistical modeling & inference

Model type and settings	N/A, see original publication
Effect(s) tested	N/A, see original publication

Specify type of analysis: ☐ Whole brain ☒ ROI-based ☐ Both

Anatomical location(s): N/A, see original publication

Statistic type for inference (See Eklund et al. 2016)	N/A, see original publication
Correction	N/A, see original publication

Models & analysis

n/a	Involved in the study
☒	☐ Functional and/or effective connectivity
☒	☐ Graph analysis
☒	☐ Multivariate modeling or predictive analysis
